# Innovative low-cost biosorption process of Cr^6+^ by *Pseudomonas alcaliphila* NEWG-2

**DOI:** 10.1038/s41598-020-70473-5

**Published:** 2020-08-20

**Authors:** Noura El-Ahmady El-Naggar, Ayman Y. El-khateeb, Abeer Abdulkhalek Ghoniem, Mohammed S. El-Hersh, WesamEldin I. A. Saber

**Affiliations:** 1 Department of Bioprocess Development, Genetic Engineering and Biotechnology Research Institute, City for Scientific Research and Technological Applications (SRTA-City), Alexandria, Egypt; 2 Department of Agricultural Chemistry, Faculty of Agriculture, Mansoura University, Mansoura, Egypt; 3 Microbial Activity Unit, Department of Microbiology, Soils, Water and Environment Research Institute, Agricultural Research Center, Giza, 12112 Egypt

**Keywords:** Biotechnology, Microbiology

## Abstract

Chromium is one of the heavy metal pollutants that causing risky health issues when discharged into the aquatic ecosystems. The current investigation focused on the bioremoval of Cr^6+^ depending on the bacterial sorption process by using *Pseudomonas* sp. NEWG-2 which was identified on the basis of morphological, cultural characteristics, 16S rRNA sequencing and phylogenetic analysis as *Pseudomonas alcaliphila* strain NEWG-2. It is clear from the FCCD experiments that the bacterium can grow normally and remove 96.60% of 200 mg/l of Cr^6+^ using yeast extract (5.6 g/l), glucose (4.9 g/l), pH (7) for 48 h incubation period. SEM and EDS analyses proved that the Cr^6+^ was biosorbed by *P. alcaliphila* NEWG-2. FTIR spectra indicated that the phenolic, carbonyl ester, acetyl, carboxylate, alkanes and carbonyl were the main groups involved in the chromium biosorption. Of the equilibrium isotherms models, the Langmuir model was more obedient, with a maximum uptake (*q*_*max*_) of 10 mg/g (bacterial-alginate beads), than the Freundlich one. The findings reveal the efficiency of *P. alcaliphila* NEWG-2 in Cr^6+^ biosorption, with feasibility in the treatment of chromium-contaminated water as a green-technology tool. Interestingly, to the best of our knowledge, this is the first report on Cr^6+^ biosorption process by *P. alcaliphila*.

## Introduction

The emerging threat of heavy metals pollution has been recognized with an adverse impact on the environment, especially in developing countries. Due to the large-scale of industrial applications, heavy metals could be considered a major health risk with biomagnified toxicity in humans, especially at the higher concentrations. Heavy metals dispersed, in free forms, in both terrestrial and aquatic ecosystems, could be extended to the human food chain, that is why, the discharge of heavy metals into the aquatic ecosystem is a concern over the last few decades^1–3^. Moreover, the non-biodegradable nature and accumulation in living organisms ensure the prolonged presence of heavy metals in the environment, and therefore great interests have been made to eradicate such ecotoxicological hazards^2,4^. On the other side, some of the heavy metals, such as Cu^2+^, Zn^2+^, Fe^2+^/Fe^3+^, in trace amounts are essential for numerous vital biological process in living organisms, like enzymatic reaction process, but at higher levels may cause extreme toxicity, owing to the inhibition of metabolic reactions of the organism^5,6^.

Owing to the rapid growth of industries (e.g. leather, textile, mining and electric manufacturing), the problem of chromium-contaminated areas has emerged^7^. Depending on the oxidation state and concentration, chromium is one of the metals that could be either constructive or lethal to the biological systems. Chromium below 100 ppm is an essential and non-toxic mineral and, further, plays a functional role in both the nucleic acid synthesis and the metabolism of lipids, glucose and amino acids^8,9^. Contrarily, another category classified chromium, especially hexavalent chromium (Cr^6+^), as toxigenic due to having a high oxidation state with exerting mutagenic and carcinogenic potential on various biological systems^10^. As stated by the WHO and Indian Standard Institution, 0.05 mg/l is the allowed level of Cr^6+^ in drinking water, while the allowable limit of Cr^6+^ in industrial effluents ranges from 2.0 to 5.0 mg/l^11^.

In the biological systems, however, most of the cellular chromium exists in the trivalent (Cr^3+^) state, compared to Cr^6+^, the Cr^3+^ form is more soluble and has stable oxidation property, and thus shows less biotoxicity. Several symptoms appear on a person contaminated by chromium, of such symptoms, nasal irritation, ulceration, skin irritation, eardrum perforation and necrosis are the most common^12^. In the environmental systems, the harmful effects extended to the number and balance of the microbial communities in various ecosystems elements, including water and soil^13^.

Environmentally, the efficient removal of heavy metals from industrial wastes, particularly from the aqueous wastes, is one of the most important concerns of the world^14^. The previously common and traditional methods applied the oxidation–reduction reactions, solvent extraction, ion exchange, chemical precipitation and adsorption for the elimination of heavy metals. These techniques are relatively expensive and ineffective, especially when the concentration of heavy metals exceeds 1 mg/l^15^.

Otherwise, biosorption is emerging as an effective eradication process of heavy metals from aqueous solutions. In which, various plants (including aquatic plants and seaweeds) and microorganisms (bacteria, fungi, yeast and algae) have been recognized as operative bio-sorbent agents^3,16–18^. The bio-removal process by microorganisms is an innovative, low cost and eco-friendly^19^. Diverse mechanisms were proposed for the microbial biosorption of heavy metals, e.g. transport across the cell membrane, biosorption by cell walls and entrapment in extracellular capsules, precipitation, complexation and oxidation–reduction reactions^5^.

The employment of bacterial biomass for metal removal treatment of wastewater is perceptively suggested by many investigators. During metal-bacteria interaction, the polarizable groups (e.g. phosphate, carboxyl, hydroxyl and amino groups) are responsible for metal binding capacity and capable of interacting with cations^20,21^. Through the removal processes by microorganisms, chromium can be eradicated during three main processes, i.e. biotransformation, bioaccumulation and biosorption, the latter is superior to other ones, with highly selective depending upon the binding capacity of biological materials used as bio-sorbents^19,22^. Interestingly, the biosorption process can take place by living and dead biomass, but the living biomass has advantages, i.e. the metal can be removed during the growth, hence eliminating the processes of microbial reproduction, drying and storage^23,24^.

Wherein, this study aimed to apply the face-centered central composite design to optimize the biosorption process of hexavalent chromium ions by varying the nutritional (yeast extract, glucose) and physical (pH and interval periods) conditions, using *P. alcaliphila* NEWG-2. The aptness of the sorption process by immobilized bacterium cells using Freundlich and Langmuir isotherms models was evaluated.

## Results and discussion

The ecosystem's healthiness and balance are greatly governed by heavy metals pollution. Chromium discharged from the effluent of the tannery and/or metal industries is a major risk and disturbance factor, in this respect^25^. That is because of the high toxicity, e.g. carcinogenic and teratogenic features, especially in the form of the hexavalent state. The annual discharged quantity of chromium into the environment is approximately 1,70,000 tonnes^26^. The relatively expensive and ineffective traditional disposal procedures motivate interests towards safe and effective alternative strategies, mainly biological-based strategy. One of such profoundly driven approach in recent times is biosorption through microbial biomass and their products^2^. The biosorption of this metal could occur during the metabolic pathways of the microorganism, which are known to survive and tolerate heavy metals stress in the contaminated environment.

Comparing to the other microorganisms, bacteria have many merits, such as the small in size, the abundant distribution, the more resistant to harsh environmental conditions, the ability to be cultured under controlled conditions, and further, possess various mechanisms of bioleaching, including bioaccumulation, biomineralization, biotransformation and biosorption^22,27^.

### Cr^6+^ tolerability of the studied bacterium

From the start point of view, the present investigation was initiated to study the tolerability of *Pseudomonas* sp. to Cr^6+^ ions, followed by optimization of the biosorption efficiency by modifying some nutritional and physical culturing conditions. Initially, K_2_Cr_2_O_7_ was used for testing the tolerability of *Pseudomonas* sp. to different Cr^6+^ ion concentrations in the batch cultured system, with running over wide ranges of initial Cr^6+^ concentrations (50–250 mg/l based on Cr^6+^ ion).

**Figure 1:**
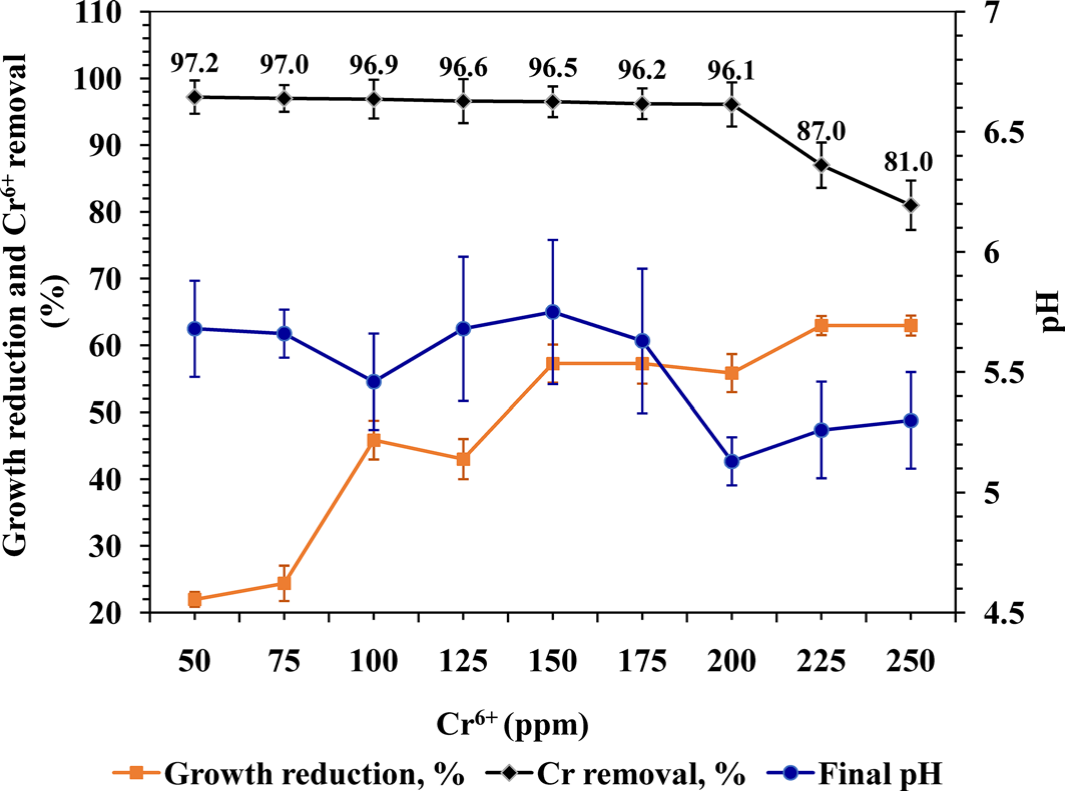
Growth reduction, Cr^6+^ removal, and final pH as a potentiality of *Pseudomonas* sp. NEWG-2 towards thresholds of hexavalent chromium.

The biosorption capacity of Cr^6+^, the bacterial growth and the final cultural pH are illustrated in Fig. 1. The bacterial biosorption capacity initiated with 97.2% at 50 mg/l, and kept around the same percent with the increasing of the initial concentration of Cr^6+^ and reached to saturated value at a concentration of 200 mg/l, with a removal efficiency of 96.1%. Higher concentrations (250 mg/l) decreased the tolerability of the bacterium down to 81%. Although the bacterium was able to grow and reduce Cr^6+^ ion content in the fermentation medium, the growth and final cultural pH slightly reduced with the increment of Cr^6+^. There was an inverse relationship between bacterial growth and final cultural pH from one side and Cr^6+^ concentrations, on the other side, this latter observation indicates the survivability and tolerability of the bacterial strain under the stress of Cr^6+^. Noticeably, *Pseudomonas* sp. was found to be able to reduce the yellow color of K_2_Cr_2_O_7_ in the medium (Supplementary Fig. 1), indicating the ability of the bacterium to grow and neutralize Cr^6+^.

Comparably, previous studies find out that some bacterial strains were tolerant up to 300 mg/l at pH 7.0 and temperature of 37 °C^28^. Nevertheless, the frequency of changes in metal uptake may be attributed to the metal's characteristics (e.g. the capacity for metal reductions, atomic weight, or ionic size ) and could be due to the characteristics of the bacterium, such as surface area, functional groups and structure^20^. In this connection, proteins and lipids may play a crucial role in the biosorption process. The capsule and slime layers of bacteria contain polysaccharides, acting as building blocks and/or barriers for heavy metal modulation. The exopolysaccharide is the self-defense of the bacterium against harsh conditions, e.g. pH, temperature, starvation and could play a crucial role in the biosorption process of metals. Likewise, Gupta and Diwan^2^ reported that the leaching of heavy metal depending on microbial extracellular polymeric substances such as polysaccharides, uronic acid, humic substances and lipids. The species of *Pseudomonas* contain exopolysaccharides with anionic functional groups, which incorporated in biosorption of heavy metals^29,30^.

On the other hand, the lower tolerability of some bacteria could be attributed to the mutagenic effect of chromium ion towards bacterial cells^31^, further, Komori et al.^32^ reported that mutation in *Enterobacter cloacae* as a result of DNA damage, leading to disruption of normal cell replication due to interactions with Cr^6+^. In some cases, the mutated cell may be more resistant to (Cr^6+^)^33^. Herein, the decline in the tolerability of *Pseudomonas* sp. NEWG-2 at the higher concentration of chromium (250 ppm of Cr^6+^) (Supplementary Fig. 1) could be due to a reduction in biosorption sites on the surface of bacteria^34^. However, it is worthy to note that the concentration applied in the present study is calculated based on Cr^6+^ ion content in K_2_Cr_2_O_7_. Consequently, the initial concentration of Cr^6+^ at 200 mg/l, has been chosen as the tolerable concentration for further investigation during the next trial.

### Identification of *Pseudomonas* sp. strain NEWG-2

The microscopic characterization of the aerobic non-spore-forming, Gram-negative; *Pseudomonas* strain NEWG-2 showed straight rods of about 1.0 to 5.0 µm long and 0.5 to 1.0 µm wide with polar flagella.

The molecular identification using 16S rRNA gene sequence analysis was employed. The obtained 16S rRNA sequence of *Pseudomonas* sp. NEWG-2 was determined and the amplified fragment gave sequence with 1,500 bp (Supplementary Fig. 2). The obtained 16S rRNA sequence was subjected to the BLAST search^35^ on the GenBank database, and the most related sequences were obtained and compared with the present sequence.

**Figure 2:**
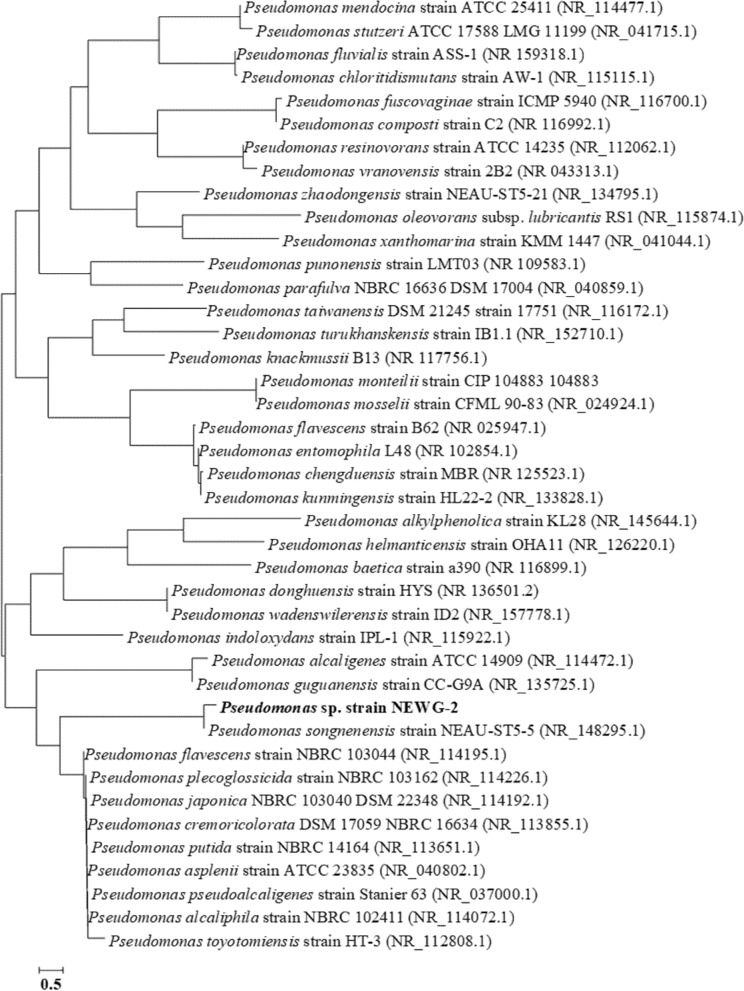
Phylogenetic tree, generated by Neighbor-Joining method with the software MEGA5, showing *P. alcaliphila* strain NEWG-2 position within the genus *Pseudomonas* based on the phylogenetic analysis of 16S rRNA genes. The bar indicates sequence divergence.

The phylogenetic tree, generated by neighbor-joining analysis (Fig. 2), was constructed using MEGA5.0 software^36^. The isolate showed a high similarity of 99.20% with *Pseudomonas alcaliphila*. Accordingly, the bacterial isolate was identified as *P. alcaliphila* strain NEWG-2. The 16S rRNA sequence had been deposited in the DDBJ/EMBL-Bank/Gen Bank database under the accession number of MN025267.

### Modeling the optimization of Cr^6+^ bioremoval process

The medium fermentation conditions of *P. alcaliphila* NEWG-2 were subjected to a statistical modulating process based on response surface methodology, i.e. the face-centered central composite design (FCCD). The FCCD of the 30-experimental runs were performed to improve the removing efficacy of Cr^6+^ ions by *P. alcaliphila* NEWG-2. The design of the matrix includes two nutritional variables, i.e. yeast extract (X_1_) and glucose (X_2_) and two physical variables, i.e. pH (X_3_) and incubation time (X_4_). The actual and coded levels of the variables as well as the investigated and predicted values of Cr^6+^ ions removal percent of the 30 runs of the FCCD matrix are displayed in Table 1.

**Table 1:** Face centered central composite design, representing Cr^6+^ removal by *P. alcaliphila* strain NEWG-2 as influenced by yeast extract, glucose, pH and incubation time.

Run			Variables				Growth (OD)	Final pH	Cr^6+^ removal percent		Residuals
Standard	No	Type	X_1_	X_2_	X_3_	X_4_			Observed	Predicted	
28	1	Center	0	0	0	0	0.397	5.26	96.03	96.29	− 0.27
20	2	Axial	0	1	0	0	0.263	5.51	95.84	95.88	− 0.04
2	3	Factorial	1	− 1	− 1	− 1	0.413	6.14	94.44	94.49	− 0.05
1	4	Factorial	− 1	− 1	− 1	− 1	0.238	6.23	93.27	93.32	− 0.05
8	5	Factorial	1	1	1	− 1	0.341	6.03	93.67	93.72	− 0.05
3	6	Factorial	− 1	1	− 1	− 1	0.233	6.08	93.45	93.43	0.02
23	7	Axial	0	0	0	− 1	0.317	6.03	95.43	95.29	0.14
30	8	Center	0	0	0	0	0.418	5.32	96.59	96.29	0.30
11	9	Factorial	− 1	1	− 1	1	0.463	4.55	93.54	93.66	− 0.12
24	10	Axial	0	0	0	1	0.296	5.84	95.42	95.59	− 0.18
7	11	Factorial	− 1	1	1	− 1	0.342	6.02	93.63	93.64	− 0.01
13	12	Factorial	− 1	− 1	1	1	0.913	4.33	94.74	94.70	0.04
29	13	Center	0	0	0	0	0.421	5.36	96.26	96.29	− 0.04
5	14	Factorial	− 1	− 1	1	− 1	0.412	5.91	93.83	93.96	− 0.13
17	15	Axial	− 1	0	0	0	0.292	5.5	95.89	95.71	0.18
14	16	Factorial	1	− 1	1	1	0.333	4.74	94.54	94.57	− 0.03
27	17	Center	0	0	0	0	0.393	5.08	96.44	96.29	0.15
26	18	Center	0	0	0	0	0.445	5.34	96.45	96.29	0.15
6	19	Factorial	1	− 1	1	− 1	0.388	6.36	94.33	94.19	0.14
22	20	Axial	0	0	1	0	0.251	5.48	95.76	95.83	− 0.07
25	21	Center	0	0	0	0	0.415	5.22	96.11	96.29	− 0.18
10	22	Factorial	1	− 1	− 1	1	0.316	5.41	94.11	94.08	0.03
4	23	Factorial	1	1	− 1	− 1	0.334	6.12	94.44	94.46	− 0.02
19	24	Axial	0	− 1	0	0	0.281	5.59	95.92	95.91	0.01
18	25	Axial	1	0	0	0	0.189	6.36	95.94	96.16	− 0.22
15	26	Factorial	− 1	1	1	1	0.382	4.55	94.70	94.66	0.04
21	27	Axial	0	0	− 1	0	0.275	5.46	95.51	95.48	0.03
12	28	Factorial	1	1	− 1	1	0.383	6.09	94.44	94.33	0.11
16	29	Factorial	1	1	1	1	0.252	5.19	94.45	94.38	0.07
9	30	Factorial	− 1	− 1	− 1	1	0.358	5.04	93.30	93.26	0.04

Variable		Coded levels									
Unit	Code	− 1	0	1							
Yeast extract (g/l)	X_1_	3	5	7							
Glucose (g/l)	X_2_	3	5	7							
pH	X_3_	6	7	8							
Incubation time (h)	X_4_	24	48	72							

Although the bacterial growth and final culture pH showed marked variation along with the 30-experimental runs of the design matrix (Table 1), their modeling and analysis failed to have significant trend and did not reveal any reasonable relationships (data not shown) and did not have any effect on the bioremoval process, therefore these data were not subjected to additional evaluation or modeling. The subsequent modeling was continued only on Cr^6+^ removal by the tested bacterium.

The variations in Cr^6+^ removal ranged from 93.30 to 96.59%. The highest level of Cr^6+^ removal percent, with a value of 96.59% (run No.8) has occurred at the center values of the tested independent variables. This observation indicates the accuracy of selected independent variables and their levels. In contrast, the minimum Cr^6+^ removal percent (93.30%) was obtained in the run no. 30, at the level of 3 g/l for both yeast extract and glucose, pH 6 and incubation time for 24 h.

### ANOVA and multiple regression analysis

To explore the fitted model, the results were subjected to multiple regression analysis (Tables 2, 3). If the value of the determination coefficient (R^2^) higher than 0.9, the regression model is described as very significant^37^. To be an adequate model, the R^2^ value should not be less than (0.75)^38^. However, the quadratic model was the best-fitted model, recording the highest values of R^2^ (0.9860), adjusted R^2^ (0.9730) and predicted R^2^ (0.9539) for Cr^6+^ removal percent using *P. alcaliphila* NEWG-2. Further, the summary of the fit statistics shows a high significance of the quadratic model (very low *P*-value < 0.0001), and non-significant lack of fit (*F*-value = 0.47 and *P*-value = 0.8541), with a small standard deviation (0.18). So, the quadratic model was selected for fitting the FCCD data of Cr^6+^ removal by *P. alcaliphila* NEWG-2 (Table 2).

**Table 2:** Fit summary for face-centered central composite design based on the design matrix of the data.

Lack of fit tests					
Source	Sum of squares	Degrees of freedom	Mean square	*F-*value	*P-*value *P*rob > *F*
Linear	31.09	20	1.55	32.62	0.0005*
2FI	29.15	14	2.08	43.68	0.0003*
Quadratic	0.22	10	0.02	0.47	0.8541
Pure error	0.24	5	0.05		

Sequential model sum of squares					
Source	Sum of squares	Degrees of freedom	Mean square	*F-*value	*P-*value *P*rob > *F*
Linear vs. mean	1.86	4	0.47	0.37	0.8263
2FI vs. linear	1.94	6	0.32	0.21	0.9694
Quadratic vs. 2FI	28.92	4	7.23	234.22	< 0.0001*
Residual	0.24	7	0.03		

Model summary statistics					
Source	Standard deviation	R^2^	Adjusted R^2^	Predicted R^2^	PRESS
Linear	1.12	0.0562	− 0.0948	− 0.3583	45.09
2FI	1.24	0.1147	− 0.3512	− 2.3923	112.60
Quadratic	0.18	0.9860	0.9730	0.9539	1.53

*Significant values, R^2^: determination coefficient, PRESS: the sum of squares of prediction error, 2FI: two factors interaction.

**Table 3:** Analysis of variance for the experimental matrix data of Cr^6+^ removal by *P. alcaliphila* strain NEWG-2 obtained by the face-centered central composite design.

Source of variance		Degrees of freedom	Sum of square	Mean of square	*F*-value	*P*-value	Coefficient estimate
Overall model		14	32.73	2.34	75.73	< 0.0001	96.29
Linear effect	X_1_	1	0.89	0.89	28.94	< 0.0001	0.22
	X_2_	1	0.00	0.00	0.16	0.6953	− 0.02
	X_3_	1	0.55	0.55	17.81	0.0007	0.17
	X_4_	1	0.42	0.42	13.49	0.0023	0.15
Interaction effect	X_1_X_2_	1	0.02	0.02	0.70	0.4155	− 0.04
	X_1_X_3_	1	0.90	0.90	29.01	< 0.0001	− 0.24
	X_1_X_4_	1	0.13	0.13	4.20	0.0584	− 0.09
	X_2_X_3_	1	0.19	0.19	6.12	0.0258	− 0.11
	X_2_X_4_	1	0.08	0.08	2.55	0.1311	0.07
	X_3_X_4_	1	0.63	0.63	20.38	0.0004	0.20
Square effect	X_1_^2^	1	0.33	0.33	10.67	0.0052	− 0.36
	X_2_^2^	1	0.40	0.40	13.05	0.0026	− 0.39
	X_3_^2^	1	1.06	1.06	34.32	< 0.0001	− 0.64
	X_4_^2^	1	1.87	1.87	60.62	< 0.0001	− 0.85
Error effect	Lack-of-fit	10	0.22	0.02	0.47	0.8541	
	Pure error	5	0.24	0.05			
R^2^			0.9860	Standard deviation			0.18
Adjusted R^2^			0.9730	Mean			94.95
Predicted R^2^			0.9539	Coefficient of variation			0.19
Adequate precision			24.38	PRESS			1.53

*Significant values, R^2^: determination coefficient, *F*: Fishers's test, *P*: probability value, PRESS: the sum of squares of prediction error.

The data were subjected to ANOVA for further exploring the aptness of the various interaction effects of the quadratic model (Table 3). However, the experimental and predicted values of chromium removal are in a decent agreement, and the value of adjusted R^2^ and predicted R^2^ are high enough to indicate a high model significance. Predicted R^2^ measures the model efficacy and significance in the prediction of new response values. The values of both predicted and adjusted R^2^ should not be ˃ 20% of each other to be in decent agreement^39^. In the present study, the predicted R^2^ is, realistically, in line with the adjusted R^2^ value, explaining the high agreement between the experimental and the predicted values of Cr^6+^ removal. The model variability (95.39%) is, thus, satisfactory enough for predicting the values of experimental variables, within the tested range.

Linear, mutual interactions or quadratic coefficient estimate with a negative value indicates that the effects of such variables are negative (antagonistic effect) on Cr^6+^ removal percent by *P. alcaliphila* NEWG-2, that is to say, an opposite association between the investigated variable (s) and the removal of Cr^6+^. While positive coefficient value means a synergistic effect and the variable(s) increase chromium removal percent by *P. alcaliphila* NEWG-2 in the investigated region of the experiment. The small value of the coefficient of variation (C.V. = 0.19%) reveals an improved accuracy and trustiness of the experiments. The value of adequate precision (24.38) is greater than 4, which is desirable and indicates the reliability of the model. The value of the sum of squares of prediction error (PRESS) is 1.53. The general mean and standard deviation values of the experimental runs are 94.95 and 1.53, correspondingly (Table 3).

The Fisher’s test (*F*-value = 75.73) and the very small value of probability (*P*-value less than 0.0001) obtained from ANOVA demonstrate that the model is very significant. Values of *P* ˃ 0.05 designate that the model term is significant^40^. When evaluating the prediction ability of the model in the Cr^6+^ removal by *P. alcaliphila* NEWG-2, the model is considered accurate with a high cogency since the statics recorded high values of adjusted R^2^ (0.973), *F*-value (75.73) and adequate precision (24.38), low values of standard deviation (0.18), coefficient of determination (0.19) and PRESS (1.53) and non-significance lack-of-fit (*P*-value = 0.8541 and *F*-value = 0.47) (Table 3).

Moreover, the significance of each parameter’s coefficient was determined by the *P*-values and *F*-value, as a rule, the parameter is significant if the *P*-value < 0.05. Based on such rule, the significant coefficients are yeast extract (X_1_), pH (X_3_) and incubation time (X_4_) as linear coefficients, X_1_X_3_, X_2_X_3_ and X_3_X_4_ as interaction coefficients and finally all the quadratic effect of the factors. On the other hand, the interactions between X_1_X_2_, X_1_X_4_ and X_2_X_4_ are not significant and not significantly contributed to the Cr^6+^ removal by *P. alcaliphila* NEWG-2.

The coefficients of regression were used to generate the equation model. The value of Cr^6+^ removal by *P. alcaliphila* strain NEWG-2 could be predicted by fitting data with the equation of the second-order polynomial in terms of the next regression model:

1 $$ \begin{aligned} Y & = + \,{96}.{29} + 0.22{\text{X}}_{{1}} - 0.02{\text{X}}_{{2}} + 0.17{\text{X}}_{{3}} \hfill \\ & \quad + 0.15{\text{X}}_{{4}} - 0.04{\text{X}}_{{1}} {\text{X}}_{{2}} - 0.24{\text{X}}_{{1}} {\text{X}}_{{3}} \hfill \\  & \quad - 0.09{\text{X}}_{{1}} {\text{X}}_{{4}} - 0.11{\text{X}}_{{2}} {\text{X}}_{{3}} + 0.07{\text{X}}_{{2}} {\text{X}}_{{4}}^{{}} \hfill \\  & \quad + 0.2{\text{X}}_{{3}} {\text{X}}_{{4}}^{{}} - 0.36{\text{X}}_{{1}}{^{{2}}} - 0.39{\text{ X}}_{{2}}{^{{2}}} \hfill \\  & \quad - 0.64{\text{X}}_{{3}}{^{{2}}} - 0.85{\text{ X}}_{{4}}{^{{2}}} \hfill \\ \end{aligned} $$

where *Y* is the value of predicted chromium removal percent, X_1_ = yeast extract concentration, X_2_ = glucose concentration, X_3_ = pH and X_4_ = incubation time.

### Model adequacy checking

**Figure 3:**
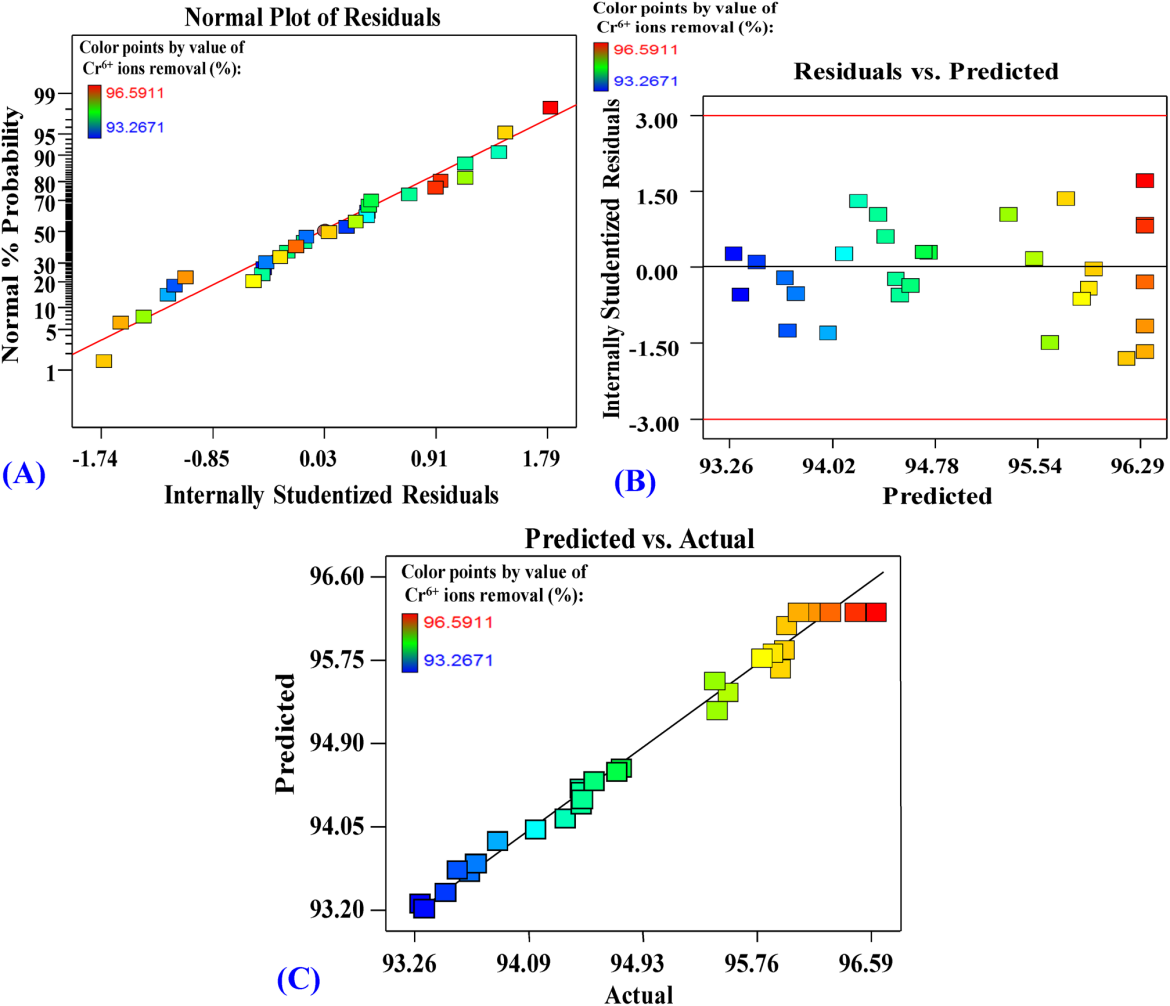
Model verification graphs, showing the normal plot of residuals (**A**), the residual against predicted values (**B**) and the experimental against predicted values (**C**) of Cr^6+^ removal by *P. alcaliphila* strain NEWG-2.

To confirm the adequacy of the model, some analytical statics were checked and depicted in Fig. 3. Plotting the normal probability of the data of the experimental residuals (Fig. 3A) shows that data points concentrated closely along the straight line, meaning that the residuals follow the normal distribution without linearity^41^. Where most of the observations gathered around the center and the values located away from the general mean and dwindled equally on both sides of the central peak. Extreme residual values on both sides are not desired. The residuals versus predicted values (Fig. 3B) was plotted, the residuals were found to be scattered randomly around the centerline and no specific patterns could be drawn, this, in turn, indicates that the residuals not correlated and distributed independently, consequently have constant variance, therefore, the model is adequately meeting the postulations of the study. Plotting the values of predicted versus actual data points (Fig. 3C) showed that the data points split equally along the 45° line. This can help detect if any value(s) cannot be easily predicted by the model. However, all data points are detectable, assuring the aptness of the model.

### Generating of the three-dimensional (3D) surface plot

**Figure 4:**
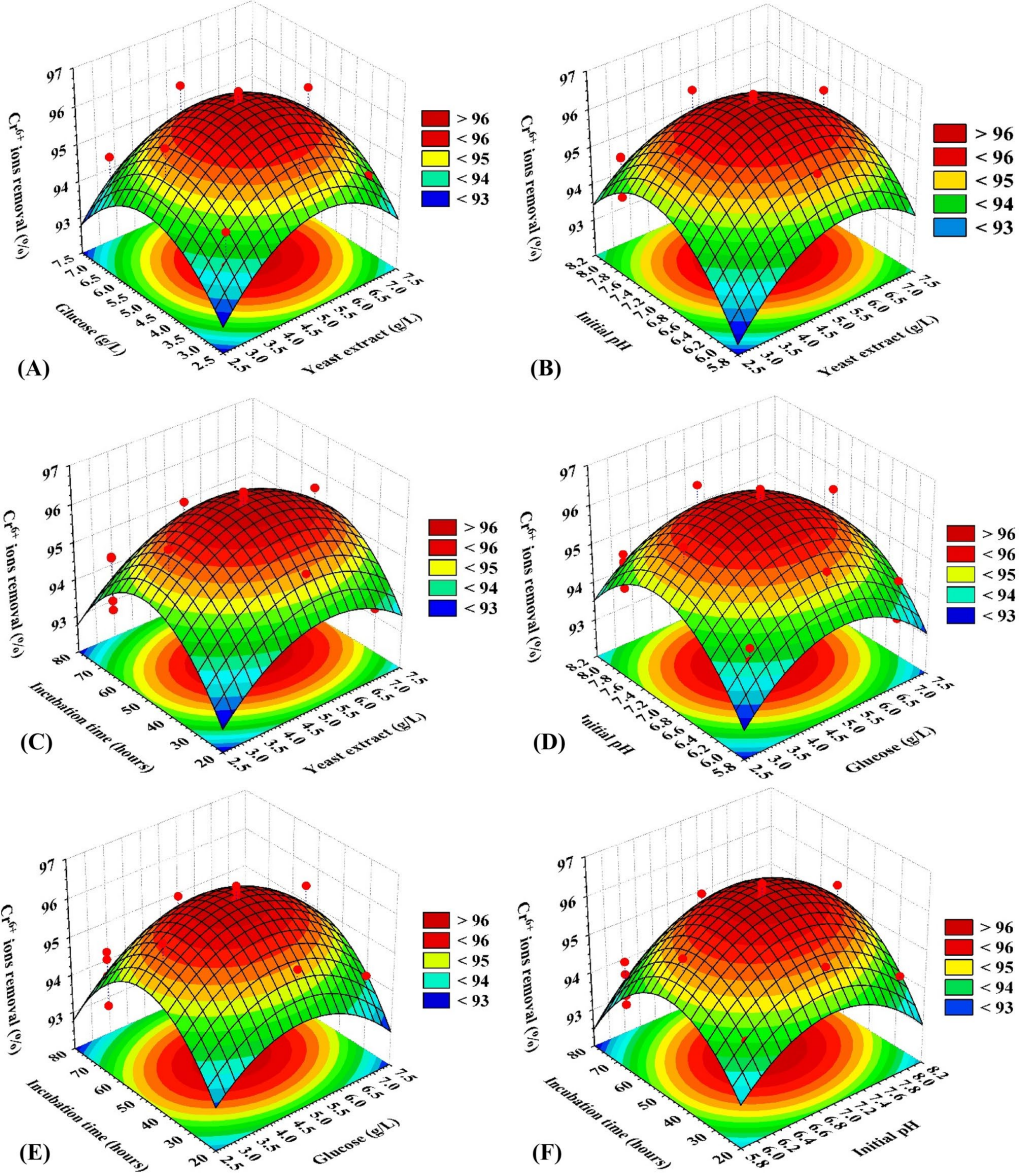
Three-dimensional surface plot of Cr^6+^ removal by *P. alcaliphila* strain NEWG-2, showing the interactive effects of each pair-wise combination of the tested variables, holding the other two variables at the center points.

Following the previous assurance of the aptness of the model, the relationship between the interaction of each couple of the tested variables and the Cr^6+^ removal was explored. For such purpose, the 3D-plots were created (Fig. 4) by drawing the two independent factors on X- and Y-axes against Z-axis (Cr^6+^ removal percent), while the other two factors are kept at the central points.

The 3D-surface plot of the simultaneous effect of yeast extract and glucose on Cr^6+^ removal was constructed (Fig. 4A). The maximum Cr^6+^ removal was located around the central points, out of this range, a rather small percentage of chromium removal was noticed. The removal percent increased with the increment of glucose concentration, but the higher-level supports a fairly low percentage of Cr^6+^ removal percent. The 3D-surface plot of Fig. 4B shows Cr^6+^ removal efficacy as a function of yeast extract concentration and pH. The Cr^6+^ removal percent maximized at pH and yeast extract around the midpoints of both. The same trend of Cr^6+^ removal was observed for the other pair-wise combinations, i.e. yeast extract and incubation time (Fig. 4C), glucose and pH (Fig. 4D), glucose and incubation time (Fig. 4E) and pH and incubation time (Fig. 4F).

### Experimental validation of the model

According to the model's Eq. (1), the highest theoretical value of Cr^6+^ removal was calculated to be 96.33% and the predicted values of the tested variable were 5.6 g/l (yeast extract), 4.9 g/l (glucose), 7 (pH) and 48 h incubation period. Under these conditions, the Cr^6+^ removal was experimentally verified and reached up to 96.60%. The experimental data verified a high grade of model precision, confirming the validation of the model under the levels of the factors used. It’s obvious that the biosorption process of chromium has been influenced by tested variables levels.

These results are comparable with the previous findings on *Bacillus* REP02, grown on various yeast extract and dextrose levels using Box-Behnken design, the predicted (98.86%) and experimentally (99.0%) values of Cr^2+^ removal were closely related^42^. Other findings reported that the maximum removal of Cr^2+^ being 94%, was occurred at pH 6.0 by coated bacteria^6^. Whereas Abhirami et al.^10^ reported 87.19% removal of Cr^6+^ at pH 4.0.

Ranjithkumar and Mahalingam^28^ stated that at pH values of 5.7 and 9, the Cr^6+^ tolerability of *Pseudomonas* sp. was up to 400 mg/l. Another data stated the significant impact of pH on the Cr^6+^ biosorption among the various variables tested using Box-Behnken design^43^. Whereas, the maximum removal of Cr^6+^ by Cyanobacteria was 81.72% at pH 11.0 and initial concentration of 15 mg/l^21^. Additionally, Durga Devi et al.^44^ investigated the biosorption of Cr^6+^ by *P. fluorescens*, the maximum uptake was found to be 800 mg/l. Other factors such as temperature, pH, redox potential and presence of other metals, play a crucial role in the chelation of chromium by *Bacillus* spp.^26^.

Importantly, the influence of medium pH values on the biosorption process could be explicated based on that the higher pH values cause the formation of metal hydroxide complexes, which decrease the concentration of chromium ions, thereby, causing a decrease in the equilibrium biosorption capacity^45^. While, a high electrostatic force of desirability at lower pH values, results in a high removal rate of chromium. Commonly, the influence of pH on the biosorption process could be due to the type of adsorbents and adsorbates^20^. Another study explored the disturbance of the bioremediation process of metals, where the building of bacterial exopolysaccharides (EPS) could be influenced by pH, in addition to other carbon sources available, temperature and the growth phase of the bacterium during which synthesis occurs^46^. Further, the strategies of heavy metal remediation through bacterial EPS must be focused on utilizing the non-neutral, negatively charged EPS to be incorporated as a suitable biosorbent. Otherwise, the bioremediation process could be influenced by nutritional factors, e.g. glucose or other carbon sources, whereby the monosaccharides form nucleotide diphosphate or monophosphate sugar, which is a crucial step in the synthesis of biosorbent agent such as EPS^47^.

In general, the microbial tolerance of heavy metal is attributed to a variety of detoxifying mechanisms, i.e. complexation by exopolysaccharides, binding with bacterial cell envelopes, metal reduction and/or metal efflux^5^. These mechanisms are sometimes encoded in plasmid genes facilitating the transfer of toxic metal resistance from one cell to another^48^. Additionally, the microbial metal tolerance can be categorized into (i) specific tolerance, which involves inducible mechanism (ii) nonspecific tolerance, which might be inducible or constitutive, some species of *Pseudomonas* fall under the nonspecific inducible resistance^49^. All these mechanisms may be applied as the suggested mode sorption process of Cr^6+^ by the present *P. alcaliphila* NEWG-2 strain.

Regarding the carbon source, glucose, d-ribose, d-xylose, d-arabinose, citrate and d-lactate were preferred as an electron donor during the metabolic process by *Acinetobacter haemolyticus*, while sucrose did not improve Cr^6+^ reduction despite enhancing the bacterium growth^50^. Whereas, *Bacillus circulans*, *Pseudomonas aeruginosa* and *Bacillus coagulans* preferred utilization of acetate, succinate, oxalate, citrate and malate as an electron donor during the metabolic process compared to glucose that needs to be catabolized into pyruvate before entering the Krebs cycle pathway^51^.

Commonly, the oxidation process of the organic compounds in synthetic media of chromium removal process might play a crucial role as an electron donor for the removal process, accumulating various types of functional groups on the surface of bacterial cells to enhance the Cr^6+^ biosorption^50^. Furthermore, the accumulation of several anionic organic compounds inside the cell induces the formation of complexes with different metal cations including chromium^26^.

Another, the reduction process of chromium by bacteria could be occurred in aerobic or anaerobic conditions, in which the aerobic reduction of Cr^6+^ is associated with a soluble protein fraction utilizing NADH or NADPH as an electron donor^52^. Whereas, in anaerobic reduction, Cr^6+^ acts as the terminal electron acceptor through membrane-bound reductase activity^53^. The species of *Pseudomonas aeruginosa*, *Pseudomonas fluorescens* and *Enterobacter cloacae* showed to be tolerant of Cr^6+^ under anaerobic conditions^33^.

### Fourier transform infrared spectroscopy (FTIR)

**Figure 5:**
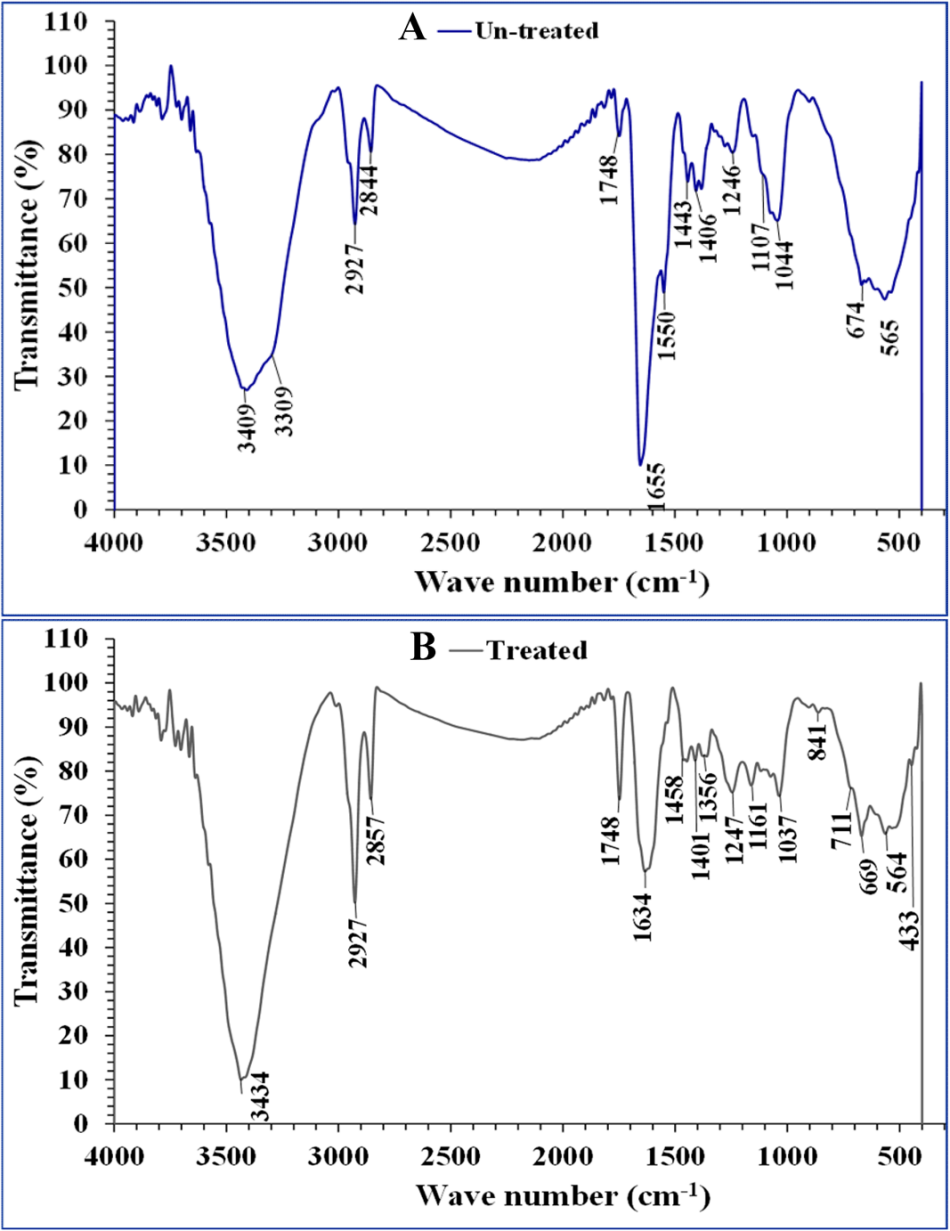
Fourier transform infrared spectroscopy analysis of *P. alcaliphila* strain NEWG-2 cells, showing the variation in bands before A: (Un-treated) and after B: (Treated) biosorption process of Cr^6+^ ion.

The FTIR spectra of *P. alcaliphila* NEWG-2 dry biomass were investigated before and after Cr^6+^ biosorption (Fig. 5). The difference in content could be returned to the interface of the metal ion with the bacterial cell wall functional groups that may include hydroxyl, carboxylate, phosphate and amino groups^54^. The ion-exchange method is the probable biosorption of metal ions on the surface of the cell. In the range of 400–4,000 cm^−1^ wave-number, the adsorption spectra were investigated^55^. The stretching vibrations of O–H at the band of 3,434 cm^−1^. The peak at 2927 cm^−1^ indicates the existence of the SP3 C–H bond. Sharp symmetric CH_2_ stretching band maximum at 2,857 cm^−1^ CH stretching at 2,844 cm^−1^. 1748 cm^−1^ for carbonyl ester group (ester C=O). The Raman band at 1,458 cm^−1^ was assigned to a scissoring vibration of CH_2_. The peaks at 1634 and 1655 cm^−1^ for C–O acetyl at lower wavenumbers. 1550 cm^−1^ strong N–O stretching. 1,458 and 1,443 cm^−1^ medium O–H bending. 1,406 cm^−1^ (COO–) carboxylate group. 1,401 cm^−1^ (C–O). 1,246 and 1,247 cm^−1^ were attributed to strong enolic C–O stretching vibration. 1107 cm^−1^ was assigned to C–O stretching, secondary alcohol. 1,161 cm^−1^ was attributed to C–O stretching’ ester. The absorption bands at 1,037 cm^−1^, 1,044 cm^−1^ assigned to the C–O–C bond. 669,674 cm^−1^ corresponding to C–Cl. Chromium oxygen (Cr–O) stretching bands have been notable at 433 cm^−1^. The lower frequency regions of IR spectra of all complexes recorded weak bands around 433–564 cm^−1^ that is attributed to Cr–N bond^56^. FTIR spectra confirmed that the phenolic, carbonyl ester, acetyl, carboxylate, alkanes and carbonyl were the main groups involved in chromium biosorption. These results are conceding with the previous investigation, which showed that the presence of different functional groups such as hydroxyl, C–H and C–N was confirmed by FTIR analysis of Cyanobacteria and *Azotobacter nigricans* NEWG-1 during removal of chromium and copper ions, respectively^3,21^.

### Scanning electron microscopy (SEM)

**Figure 6:**
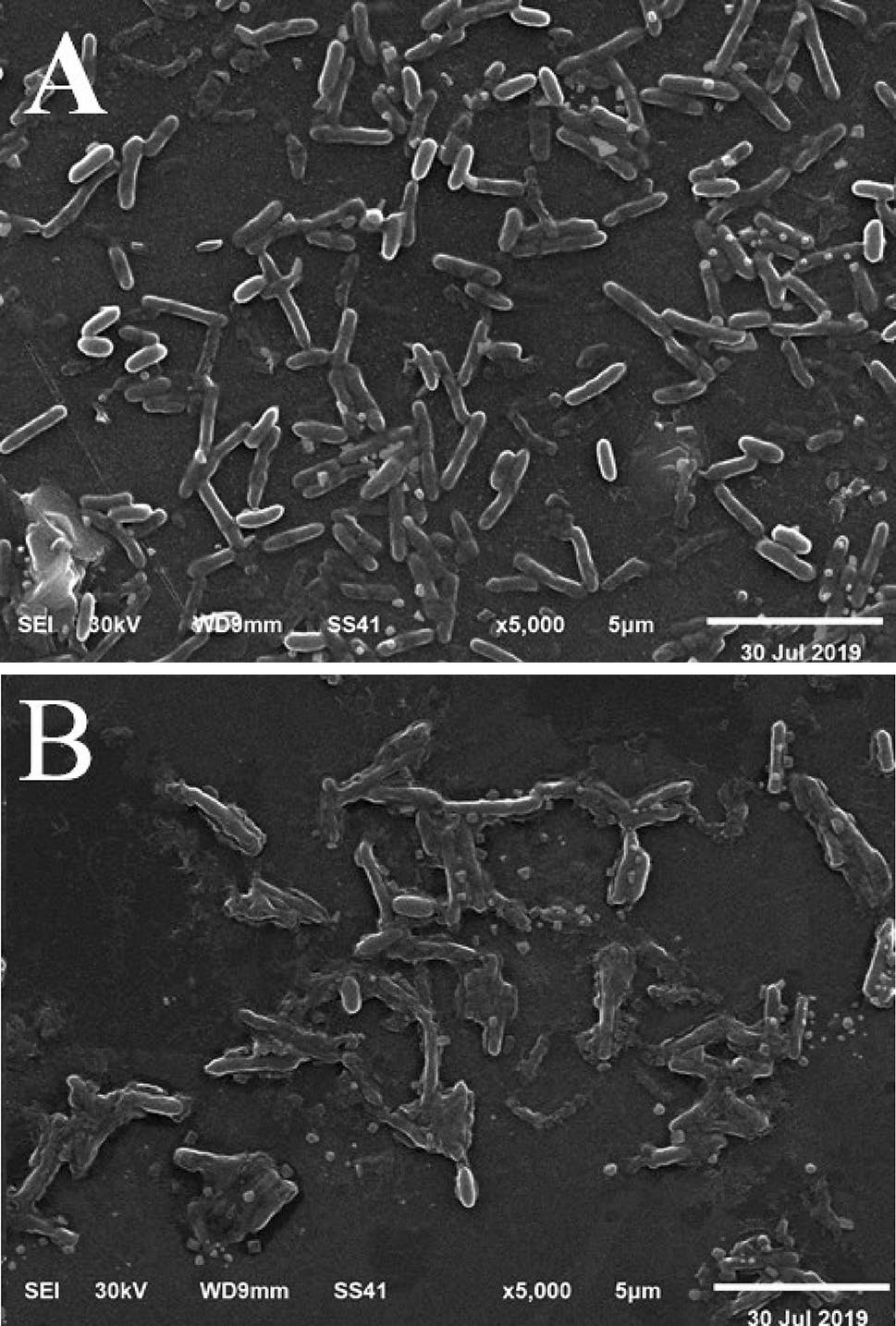
Micrograph of scanning electron microscopy, showing the normal cells of *P. alcaliphila* strain NEWG-2 (**A**) and the alteration (**B**) occurs after biosorption of Cr^6+^ ions.

SEM data that recorded the morphological variation in *P. alcaliphila* NEWG-2 before and after the biosorption process of Cr^6+^ are depicted in Fig. 6. The scanning electron micrograph clearly illustrates the surface texture and morphology of *P. alcaliphila* NEWG-2 with a large magnification. The SEM analysis revealed also a piece of crucial information on the surface morphology of bacterium, with more extension. The graph indicates, obviously, the difference between the micrographs before and after adsorption of Cr^6+^ ions.

### Electron dispersive spectroscopy (EDS)

**Figure 7:**
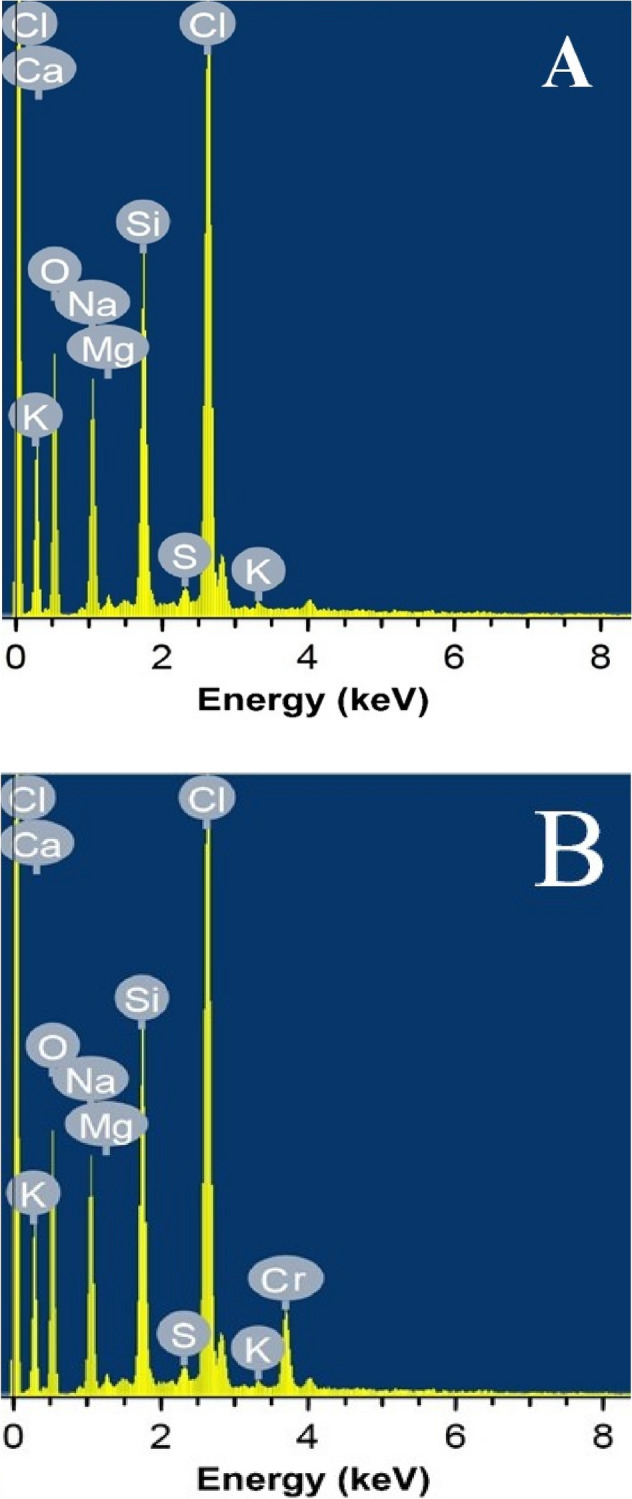
Analysis of electron dispersive spectroscopy of *P. alcaliphila* strain NEWG-2, showing the normal cell elements before (**A**) treatment in comparison to the emerging peak of Cr^6+^ ion after (**B**) the biosorption process.

One useful tool, for analysis of elemental content and/or chemical characterization of sorbents, is the EDS^57^. Herein, the EDS analysis was studied to investigate how well the Cr^6+^ attached to the cell wall of *P. alcaliphila* NEWG-2 biomass. Figure 7 depicts the presence of additional Cr^6+^peak after the biosorption process by *P. alcaliphila* NEWG-2 and these data confirmed the ability of this bacteria to remove Cr^6+^ ions from aqueous effluents.

### Isotherms of Cr^6+^ biosorption by immobilized cells

Biosorption equilibrium isotherm modeling of alginate beads alone or immobilized with *P. alcaliphila* NEWG-2 was studied for Cr^6+^. Data of initial and residual concentrations of Cr^6+^ after 4 h of direct contact at 25 °C, as well as, the summary of constants for both Langmuir and Freundlich's constants are presented in Table 4. Initially, the lower the value of *b* constant of the Langmuir model and the higher the values of both *K*_*f*_ and *n* constants of the Freundlich model, indicate directly the higher affinity of the biomass.

**Table 4:** Initial and final Cr^6+^ concentrations and the estimated Langmuir and Freundlich constants after 4 h contact with alginate beads with or without bacterial inoculation at 25 °C.

*C*_0_ (initial Cr^6+^, mg/l)	*C*_e_ (final Cr^6+^ or equilibrium, mg/l)	
	Inoculated beads	Uninoculated beads
200	3.404	35.636
250	5.910	49.343
300	10.076	98.350
350	15.453	119.474
400	31.554	137.114
**Langmuir**		
*q*_*max*_(mg/g)	10.000	7.405
*b* constant (L/mg)	0.2468	0.0334
R^2^	0.9990	0.9225
R_L_ (equilibrium type)	0.01002 (favorable)	0.069
**Freundlich**		
*K*_*f*_ constant (mg/g)	21.337	3.06196
*n* constant	3.652	3.7232
R^2^	0.9180	0.74432

**Figure 8:**
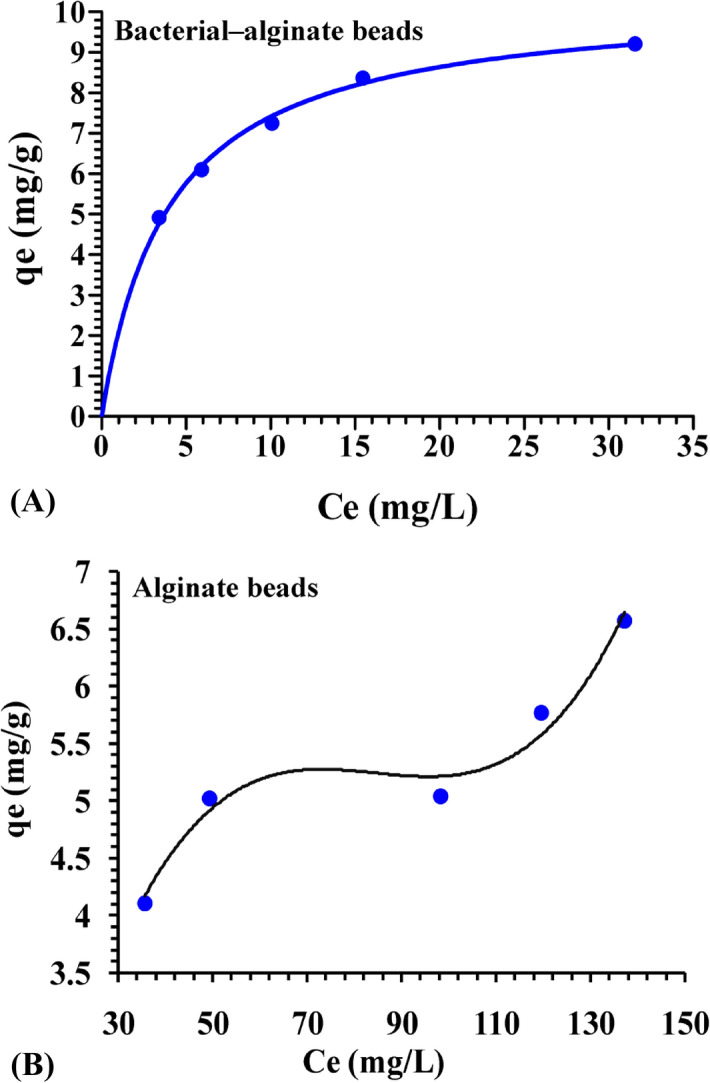
Biosorption of Cr^6+^ ions by alginate beads inoculated (**A**) or uninoculated (**B**) with *P. alcaliphila* NEWG-2 as a function of initial concentration. *q*_*e*_ is the amount of Cr^6+^ adsorbed at the equilibrium; *C*_*e*_ is the residual concentration of Cr^6+^.

**Figure 9:**
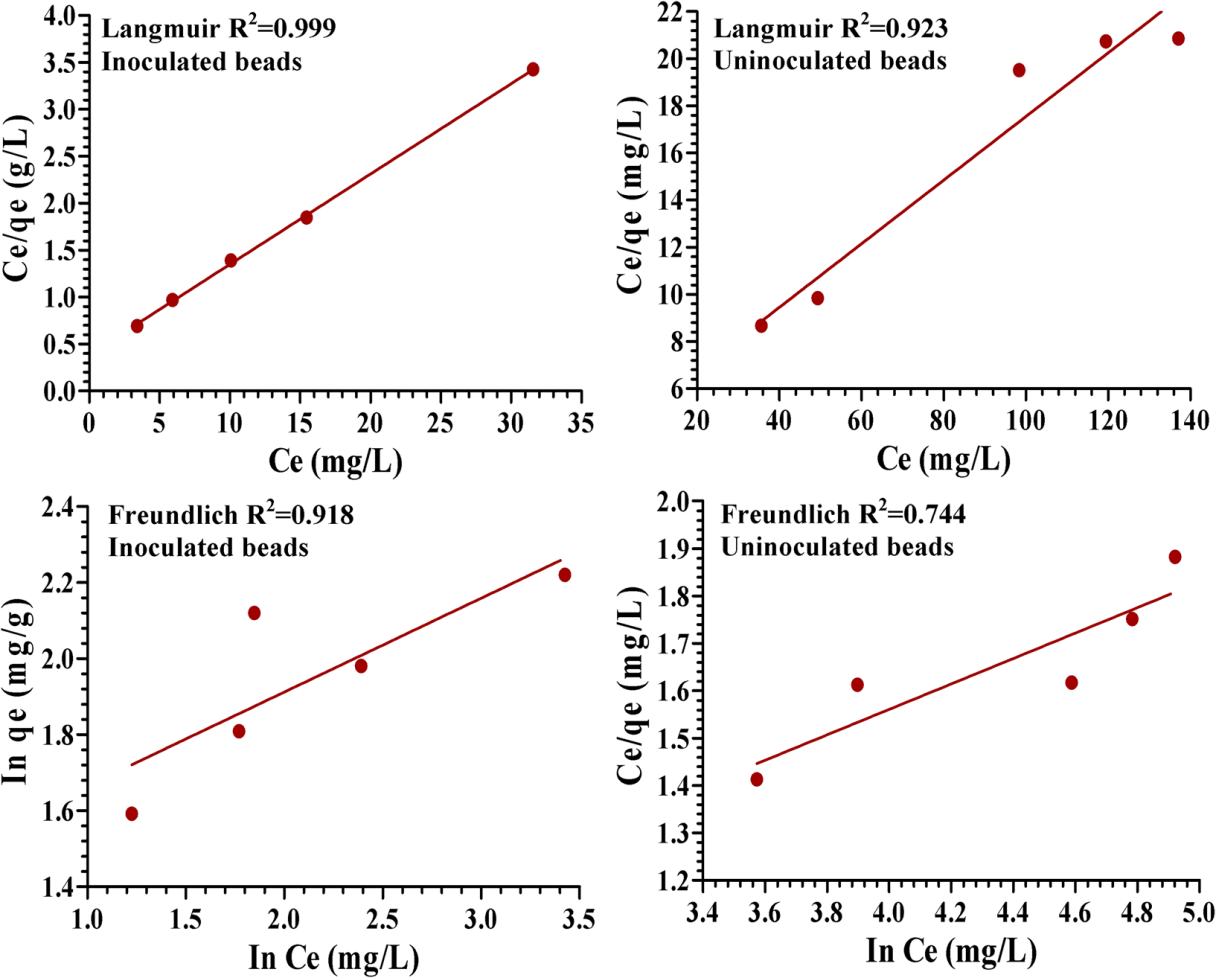
Linear plots of Langmuir and Freundlich models for the adsorption of Cr^6+^ by the immobilized *P. alcaliphila* NEWG-2 in alginate beads and uninoculated-alginate beads. *q*_*e*_ is the amount of Cr^6+^ adsorbed at the equilibrium; ln *q*_*e*_ is the natural logharitm of the amount of Cr^6+^ adsorbed at the equilibrium; *C*_*e*_ is the residual concentration of Cr^6+^; ln *Ce* is the natural logharitm of the residual concentration of Cr^6+^.

The amount of metal adsorbed at the equilibrium (*q*_*e*_) versus residual concentration (*C*_*e*_) sorption isotherm relationship was mathematically expressed and plotted (Fig. 8). The value of *q*_*max*_ of bacterial-alginate beads was 10 mg/g compared with only 7.405 mg/g for alginate beads. This, in turn, reflecting the critical role of the immobilized cells in the biosorption process. The linear regressions of data for the Langmuir and Freundlich isotherms for Cr^6+^ biosorption using immobilized *P. alcaliphila* NEWG-2 cells were plotted in Fig. 9. The equilibrium parameter (R_L_) or the dimensionless constant separation factor is essentially used to express the Langmuir isotherm, which indicates the shape of the isotherm adsorption for the adsorbents and adsorbates. In this respect, R_L_ has various types, i.e. favorable (0 < R_L_ < 1), linear (R_L_ = 1) and, unfavorable (R_L_ > 1) the present value (0.01002) at different initial concentrations of Cr^6+^ for the Langmuir model indicates a favourable adsorption process. The coefficients of determination (R^2^) of both Langmuir and Freundlich are close to 1.0, i.e. 0.999 and 0.918, respectively, therefore, adequately, describing the experimental data of Cr^6+^ biosorption. The higher the R^2^ the more robustness of the model. Langmuir model was found to be more efficient in this respect.

Likewise, the biosorption process in alginate beads only (Fig. 9) was apt to Langmuir model than Freundlich, in which the R^2^ is 0.9225 and 0.7443, respectively. Briefly, the immobilization of the bacterial cells made a difference in the biosorption process and such process is more obey to Langmuir model than Freundlich’s one.

The current data are inconsistent with the findings of Rezaei^58^, in which the equilibrium isotherm models of the sorption process of Cr^6+^ by *Spirulina* sp. preciously followed Freundlich than Langmuir. Various equilibrium models for fitting the experimental data for examining the correlation between solid and liquid phase concentrations of Cr^6+^ at equilibrium had been investigated^21^. In common, the two major models of kinetic study, i.e. Langmuir and Freundlich isotherms, were the major models^59^, in which, these mathematical models can fit the data reasonably well, and hardly reflect the sorption mechanism.

## Materials and methods

### Bacterium and growth conditions

*Pseudomonas* sp. was isolated, 2 years ago, from the soil and identified at the genus level by the Department of Microbiology, Soils, Water and Environment Research Institute, Agricultural Research Center (Affiliation ID: 60019332), Giza, Egypt. The isolate was kindly provided to the present study.

The fermentation medium consisting of (g/l) glucose (5), yeast extract (5) and MgSO_4_·7H_2_O (0.2) and pH 7.2. For the maintenance, the bacterial strain was cultured on slants of the same medium supported with 15 g agar and incubated at 25 ± 1 °C for 48 h, the autoclavation was carried out at 121 °C for 20 min. the bacterium was sub-cultured periodically and preserved at 4 °C.

For inoculum preparation, the bacterium was grown on the previous broth medium under shaking at 100 rpm and 25 ± 1 °C for 48 h, the bacterial count was adjusted to obtain 10^8^ ml^−1^ CFU, 5% (v/v) inoculum was used to inject 50 ml of fermentation medium Erlenmeyer flasks.

### Bacterial tolerance to Cr^6+^

The potentiality level of *Pseudomonas* sp. NEWG-2 to survive in Cr^6+^ was investigated. The preceding fermentation medium was supplemented with various concentrations of K_2_Cr_2_O_7_ to cover a range from 50 to 250 ppm of Cr^6+^, and incubated for 48 h. Then the bacterial tolerability was evaluated in terms of growth reduction, Cr^6+^ removal after fermentation and a reduction in the final cultural pH.

### Molecular identification

*Pseudomonas* sp. NEWG-2 was molecularly identified using 16S rRNA sequencing. DNA extraction for the bacterial sample was performed using the Thermo Gene JET Genomic DNA Purification Kit (#K0721), followed by the PCR reaction and sequencing according to the method of El-Naggar et al.^60^. The universal primers; 27F (5′-AGAGTTTGATCCTGGCTCAG-3′) and 1492R (5′‐TACGGYTACCTTGTTACGACTT‐3′) were used. The phylogenetic tree was constructed using the Neighbor-Joining method with the software MEGA5. The bar indicates sequence divergence^61^.

### Statistical optimization of Cr^6+^ biosorption

The matrix of FCCD was performed for statistically modeling of the medium conditions for the maximization of the bioremoval process of Cr^6+^ by *P. alcaliphila* NEWG-2. Two nutritional (yeast extract and glucose) and two physical (pH and incubation time) factors were the independent factors used for constructing the design matrix. Each of the four factors was investigated at three levels during the fermentation (Table 1). The FCCD was arranged to include eight axial points, sixteen factorial points, six center points and to estimate the pure error^62^. Following the laboratory experimentation of the design, the fermented medium was centrifuged (5,000 rpm for 20 min), the biosorption process was evaluated.

### Evaluation of the biosorption process

The efficacy of bacterial isolate to survive in the Cr^6+^-containing medium was evaluated, measuring the bacterial growth (OD at 610 nm) and the ability to modify the fermentation medium pH (using pH-meter with a glass electrode (HI 9,321 microprocessor pH-meter). Finally, the residual Cr^6+^ was determined in the supernatant after fermentation. The concentration was recorded on a Buck Scientific Accusys 211 series, Atomic Absorption Spectrophotometer, USA by an air/acetylene flame system^63^.

### Data modeling

The obtained data were then fitted to the next second-order polynomial quadratic model:

2 $$\text{Y} = {\upbeta}_{0} +{\sum}_{\text{i}}{\upbeta}_{\text{i}}{{\text{X}}}_{\text{i}} + {\sum}_{\text{ij}}{\upbeta}_{\text{ij}}{{\text{X}}}_{\text{i}}{{\text{X}}}_{\text{j}} + {\sum}_{\text{ii}}{\upbeta}_{\text{ii}}{{\text{X}}_{\text{i}}}^{2}$$

where; Y is the Cr^6+^ removal percent, X_i_, and X_j_ are independent variables; β_0_ model constant, β_i_, is linear coefficients; β_ii_ is the quadratic coefficients and β_ij_, is the interaction coefficients. To evaluate the equation model, laboratory validation was performed to ensure the fitness of the theoretically calculated value of each factor.

### FTIR spectroscopy

Before and after Cr^6+^ removal, the cells of *P. alcaliphila* NEWG-2 were analyzed using FTIR spectroscopy to detect the functional groups present in the cells. With KBr pellets, the bacterial cells were implemented. The FTIR spectra of *P. alcaliphila* NEWG-2 were estimated in the range from 400 to 4,000 cm^−1^ (Thermo Fisher Nicolet IS10, “USA spectrophotometer) at Spectral Analyses Unit, Mansoura University, Egypt^3^.

### SEM investigation

To evaluate the removal of the Cu^2+^ and to examine the surface of bacterial cells, dry cells of *P. alcaliphila* NEWG-2 (before and after removal of the Cr^6+^) were gold-coated and examined at various magnifications using SEM (JEOL TEM-2100) attached to a CCD camera at an accelerating voltage of 200 kV at Central Laboratory, Electron Microscope Unit, Mansoura University, Egypt^3^.

### EDS evaluation

Energy-dispersive X-ray analysis was performed, employing JEOL TEM-2100 connected to a CCD camera at an accelerating voltage of 200 kV at Central Laboratory, Electron Microscope Unit, Mansoura University, Egypt^3^.

### Bacterial immobilization

Immobilization of *P. alcaliphila* NEWG-2 cells was performed in 4% sodium alginate in distilled water, prepared by constant stirring at 60 °C for 30 min^64^. Following cooling, the sterile sodium alginate gel was supplied with bacterial cells (10^5^ CFU/ml from 48 h old culture) with stirring for 5 min at room temperature. The beads with a diameter of 1.5 ± 0.2 mm were generated by adding drop-wise of the alginate alone or alginate-bacterial biomass combination in a cold sterile solution of CaCl_2_ (2.5%) through a 3-ml syringe with gentle stirring at room temperature to make spheres beads, then, by washing, three times with distilled sterilized water, the trace of CaCl_2_ was removed and finally, stored at 4 °C overnight in sterilized distilled water for stabilization and hardness. For biosorption procedure, glass separating funnel (Simax) was packed with the alginate-bacterial beads or alginate beads without bacteria. Initial concentrations ranging from 200 to 400 ppm of Cr^6+^ ion solution were added and left at 25 °C under shaking (100 rpm). Samples of each concentration from both treatments were collected after 4 h from the separating funnel effluent. The collected fractions were analyzed for the residual ions with the Atomic Absorption Spectrophotometer (Buck scientific 210 VGP, Inc.).

### Equilibrium isotherms

The biosorption equilibrium uptake capacity in the absence or presence of the bacterial cells in sodium alginate beads at each concentration of Cr^6+^ was calculated according to mass balance on the ions expressed using the next equation:

3 $$q_e = \frac{\left({C}_{0}  - {C}_{e}\right)}{M} \times V$$

where *M* is the biomass dry weight (g), *C*_*e*_ is the concentration of Cr^6+^ (mg/l) at equilibrium, *C*_0_ is the original Cr^6+^ concentration (mg/l),* V* is the sample volume (liter), and *q*_*e*_ is the biomass biosorption equilibrium ions uptake capacity (mg/g).

Freundlich's and Langmuir isotherms were used to characterize the equilibrium between adsorbed ions by the bacterial cells and ions in the solution in this study. The equation of Langmuir isotherm empirical model based on the sorption on the surface is as follows:

4 $${q}_{e} = \frac{{q}_{max}C_{e}b}{1 + C_{e}b}$$

Then after arrangement, the next equation is generated;

5 $$\frac{C_{e}} {q_{e}}=\frac{1} {q_{max}{b}}+\frac{C_{e}} {q_{max}}$$

The *b* and *q*_*max*_ values (the adsorption equilibrium constants) can be obtained from the intercepts and the slopes of the linear plots; respectively, where experimental data of *C*_*e*_/*q*_*e*_ as the function of *C*_*e*_.

### Freundlich isotherm model

The equation of Freundlich isotherm empirical model based on sorption on a heterogeneous surface is as follows:

6 $${q}_{e}=  {K}_{f} {({C}_{e})}^{n}$$

*K* and *1/n*: an experimental constant, *n* represents the concentration effect on the adsorption efficiency and reflects the adsorption strength. *K* means the adsorption capacity of the adsorbent. The equation can be linearized in the following logarithmic form:

7 $$\ln{q}_{e} =\ln \, {K}_{f}+ \frac{1}{{n}} \, \ln{C}_{e}$$

These values *K*_*f*_ and n can be obtained from the intercepts and the slopes of the linear plots; respectively, where experimental data of ln *q*_*e*_ as the function of ln *C*_*e*_.

### Experimental design and statistical analysis

The matrix of FCCD and statistical analysis of variance (ANOVA) were performed using the statistical software packages Minitab (version 18, Minitab Inc., U.S.A.) and Design-Expert (version 7, Stat-Ease, Minneapolis, USA). The STATISTICA software (Version 8.0, StatSoft Inc., Tulsa, USA) was employed for plotting the 3D-surface plots. The Origin 2018 software (Version 2018, OriginLab Corporation, Northampton, USA) was used for analysis and graphing of isotherms. Experiments were accomplished in triplicates.

## Conclusions

The present investigation concluded the optimization process for biosorption of Cr^6+^ using statistical modeling; FCCD. The maximum removal of Cr^6+^ (96.60%) by *P. alcaliphila* strain NEWG-2 was achieved in a medium containing yeast extract (5.6 g/l) and glucose (4.9 g/l) with an initial pH (7) and fermented for 48 h. The immobilized cells showed also efficacy in the biosorption process of Cr^6+^ following Langmuir and Freundlich isotherms model. The equilibrium data were compliant to Langmuir isotherm model compared to the other one. Finally, the current new green tool technology; *P. alcaliphila* NEW-2 is recommended to be the biosorption of chromium from the contaminated water as a safe and cost-effective strategy.

## Supplementary information

Supplementary Information 1.

Supplementary Information 2.
