# Supplementary material for: Innovative low-cost biosorption process of Cr6+ by Pseudomonas alcaliphila NEWG-2
Source: Sci Rep. 2020 Aug 20;10:14043. doi: 10.1038/s41598-020-70473-5 (PMC7441394; doi:10.1038/s41598-020-70473-5)
Supplement: Supplementary file 1 — Supplementary Information 1. [file 41598_2020_70473_MOESM1_ESM.docx]

**Innovative low-cost** **biosorption process of Cr**^6+^ **by** ***Pseudomonas* *alcaliphila* NEWG-2**

**Noura El-Ahmady El-Naggar^1^, Ayman Y. El-khateeb^2^, Abeer Abdulkhalek Ghoniem^3^, Mohammed S. El-Hersh^3^, WesamEldin I.A. Saber^3^**

^1^Department of Bioprocess Development, Genetic Engineering and Biotechnology Research Institute, City for Scientific Research and Technological Applications, Alexandria 21934, Egypt.

^2^Department of Agricultural Chemistry, Faculty of Agriculture, Mansoura University, Egypt.

^3^Microbial Activity Unit, Department of Microbiology, Soils, Water and Environment Research Institute, Agricultural Research Center, P.N. 12112, Giza, Egypt.

**Corresponding Author’s information**

**Dr. Noura El-Ahmady Ali El-Naggar**

**Address:**

Bioprocess Development Department,

Genetic Engineering and Biotechnology Research Institute,

City of Scientific Research and Technological Applications,

New Borg El- Arab City, 21934, Alexandria, Egypt

**Tel:** (002)01003738444

**Fax:** (002)03 4593423

**E-mail:** [nouraalahmady@yahoo.com](mailto:nouraalahmady@yahoo.com)


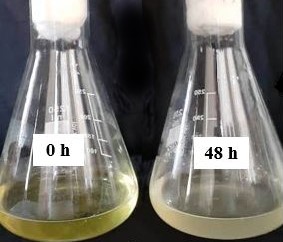


**Supplementary Figure 1.** Fermentation flasks showing the efficiency of *Pseudomonas* sp. in discoloration of the yellow color of K_2_Cr_2_O_7_ after 48 h of fermentation compared to the control (0 h)

**Supplementary Figure 2.** Agarose gel electrophoresis showing the PCR product of the amplified 16S rRNA fragment of the strain; *Pseudomonas alcaliphila* NEWG-2*.*
